# Review of the clinical spectrum of Fahr’s syndrome

**DOI:** 10.1192/j.eurpsy.2022.1635

**Published:** 2022-09-01

**Authors:** L. Garcia-Mendaza, Á. Ruiz De Pellón

**Affiliations:** Hospital Universitario Donostia, Psychiatry, Donostia, Spain

**Keywords:** Fahr’s syndrome, Fahr’s disease, calcification of basal ganglia

## Abstract

**Introduction:**

70-year-old male with previous diagnosis of bipolar disorder and poor adherence. In the past months, he showed incipient behavioral alterations, for which he entered psychiatry service. During adressment, frequent memory failures, isolation, apathy and mutism were identified, clasifying the case as a possible dementia. A CT-scan was performed, revealing bilateral, simetrical calcifications of the basal ganglia, compatible with Fahr’s syndrome.

**Figure:**
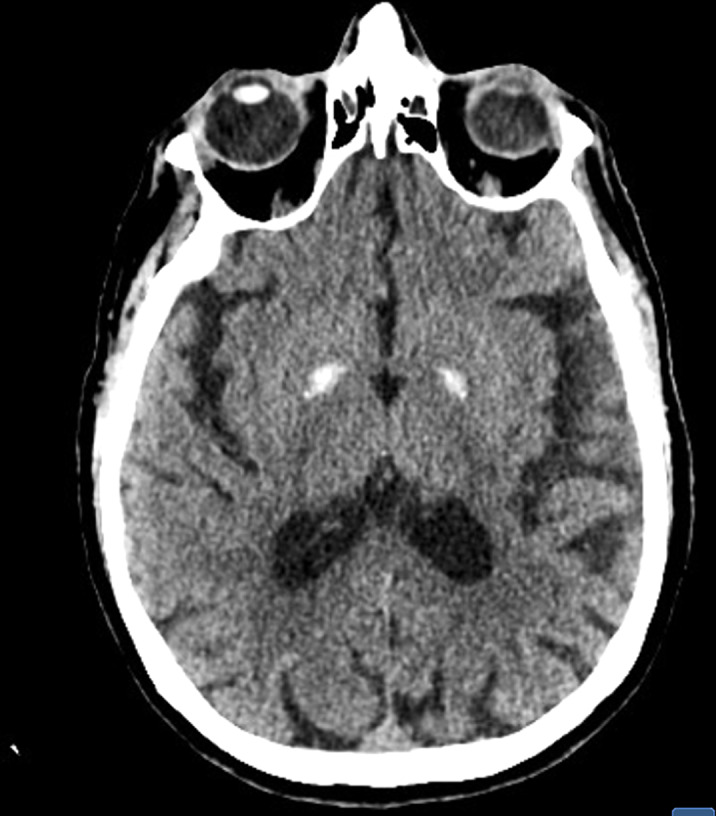

**Objectives:**

Review of the available literature regarding Fahr’s syndrome, a rare condition witch can lead to a wide spectrum of neurological, motor and behavioral symptoms.

**Methods:**

A bibliographic revision has been carried out. Sources used: Google scholar, PubMed.

**Results:**

Fahr’s syndrome is characterized by symmetric and bilateral calcification of the basal ganglia, as well as other areas related to motor functions, such as the cerebellum. It is believed that it has an autosomal dominant inheritance, and the symptoms appear between the ages of 40 and 60. The spectrum of clinical manifestations includes motor disorders such as parkinsonism or chorea. The appearance of dementia or psychiatric disorders, such as schizophrenia like psychosis or mood disorders, are also common. Also pyramidal symptoms, cerebellar dysfunction, speech difficulty or convulsive seizures can be identified.

**Conclusions:**

Fahr’s syndrome is rare, with a prevalence of <1 / 1,000,000. Diagnose is based on a compatible CT-scan, with clinical features and exclusion of other medical conditions. Nowadays, treatment is limited to a symptomatic support. The goals of further research are to understand the genetics of this disorder which could lead to an effective method for treating and preventing Fahr’s syndrome.

**Disclosure:**

No significant relationships.

